# Supplementary material for: Dynamic intramolecular regulation of the histone chaperone nucleoplasmin controls histone binding and release
Source: Nat Commun. 2017 Dec 20;8:2215. doi: 10.1038/s41467-017-02308-3 (PMC5738438; doi:10.1038/s41467-017-02308-3)
Supplement: Supplementary file 1 — Supplementary Information [file 41467_2017_2308_MOESM1_ESM.pdf]

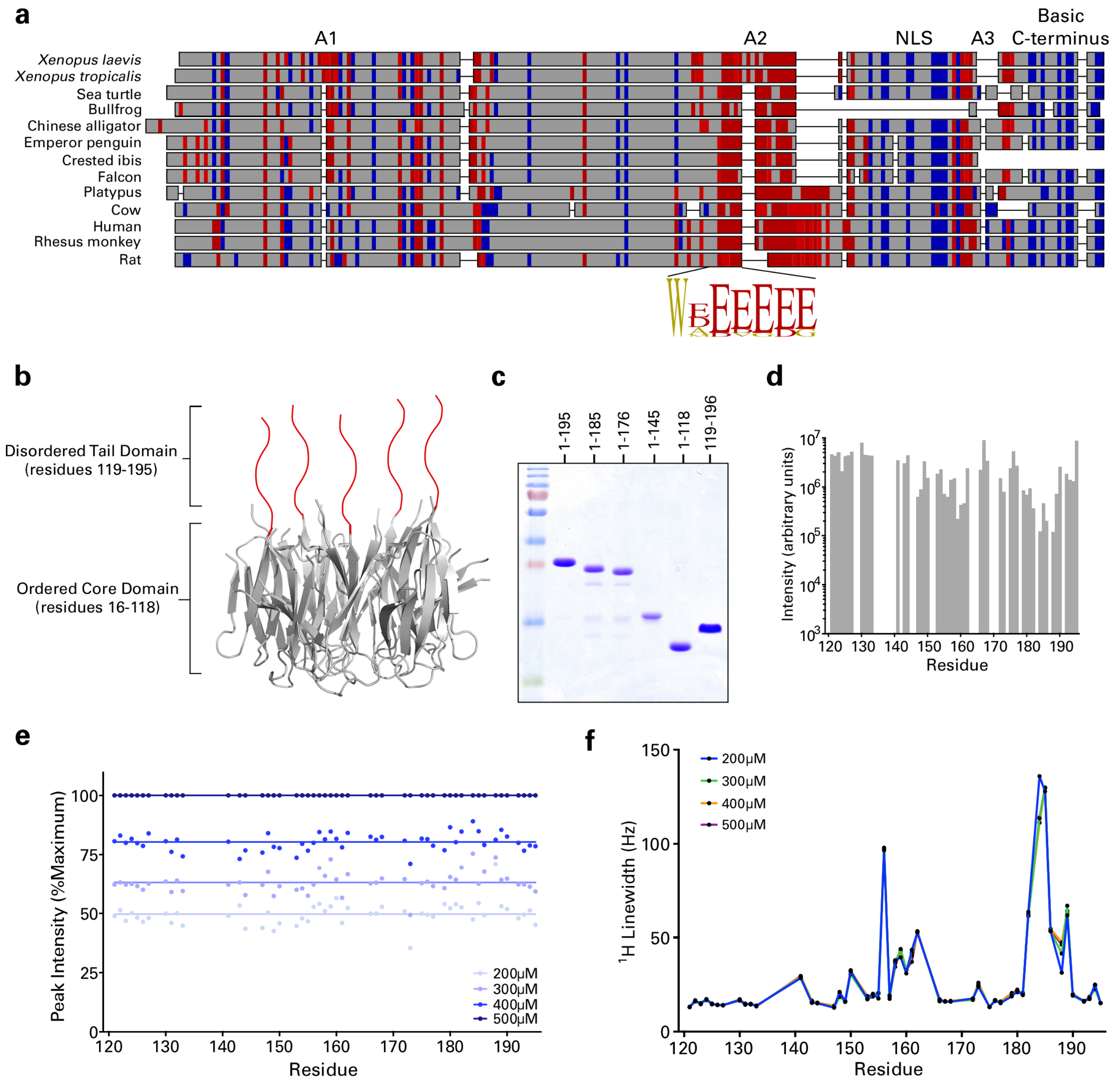


## Supplementary Figure 1: Npm Sequence Alignment and Truncations

a. Sequence alignment of Npm from various vertebrates highlighted by charge (red = acidic, blue = basic). Conservation analysis sequence logo of residues 125-131.

b. Relative orientation of the two domains of Npm. Grey=pentameric Core domain (PDB 1K5J), Red=Disordered Tail domain schematic.

c. Recombinant Npm truncations run on a 15% SDS-PAGE and stained with Coomassie Blue.

d. Relative intensities (log scale) of assigned peaks in the Tail domain HSQC. Significantly less intense peaks observed between residues 155-162 and 179-189 suggestive of conformational exchange.

e. Concentration dependence in peak intensities derived from HSQC spectra of the Tail domain taken from 200-500μM normalized to maximum intensity of each peak. Stepwise increases in peak intensities as a function of concentration are consistent with little to no self-association.

f. Concentration dependence in $^1$H linewidths derived from HSQC spectra of the Tail domain taken from 200-500μM. Broadened peaks from residues 155-162 and 179-189 is independent of concentration, consistent with little to no self-association

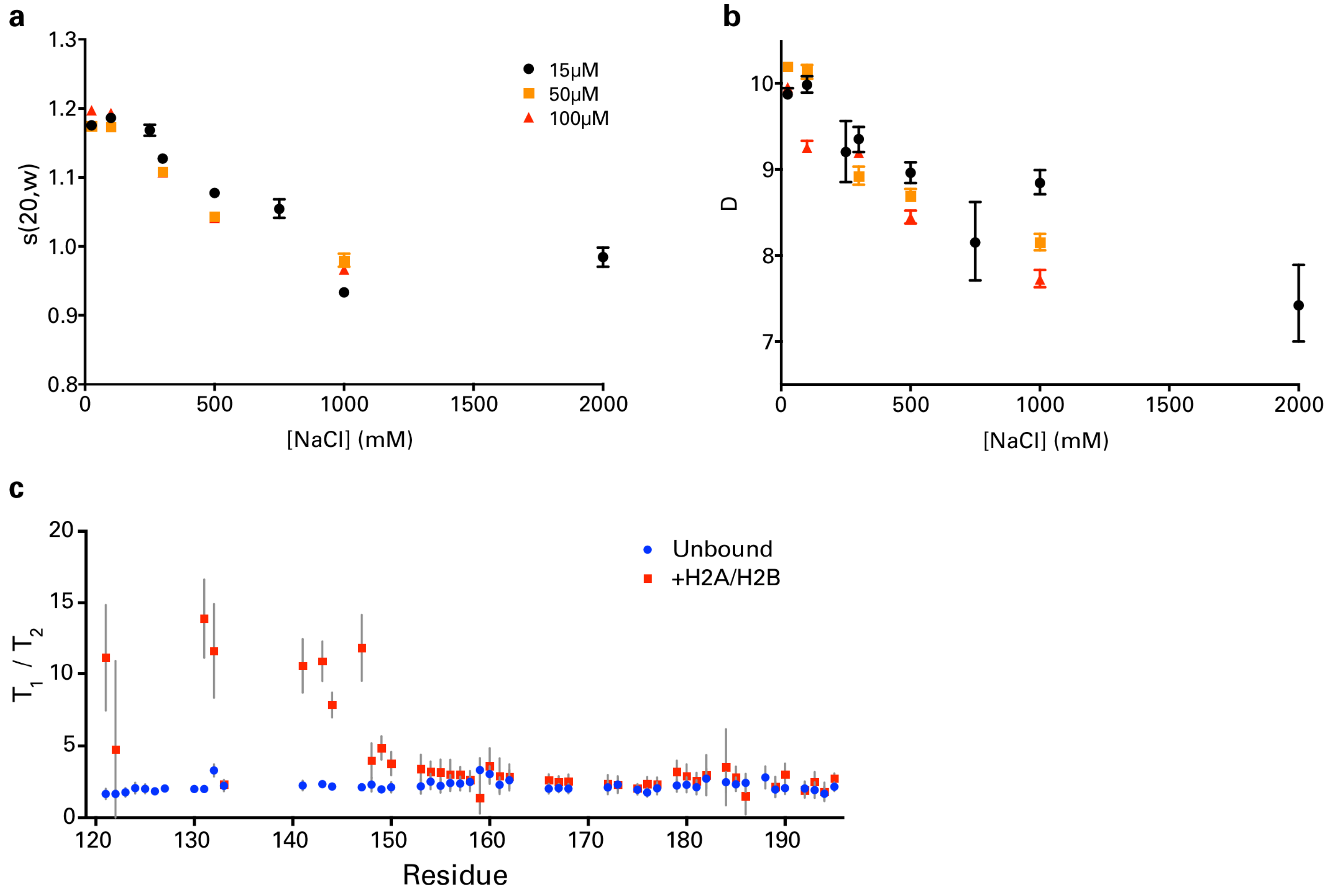


**Supplementary Figure 2: Biophysical Analyses of the Npm Tail Domain**

a. Sedimentation coefficient values of the Tail domain at 3 different concentrations (15, 50 and 100μM) at increasing [NaCl].
b. Diffusion coefficient values of the Tail domain at 3 different concentrations (15, 50 and 100μM) at increasing [NaCl].
c. Ratio of T1 to T2 relaxation times for the Tail domain in the unbound (blue circles) and H2A/H2B-bound (red squeares) states. Significant increases in T1/T2 upon binding were observed for assigned residues between 121-149.

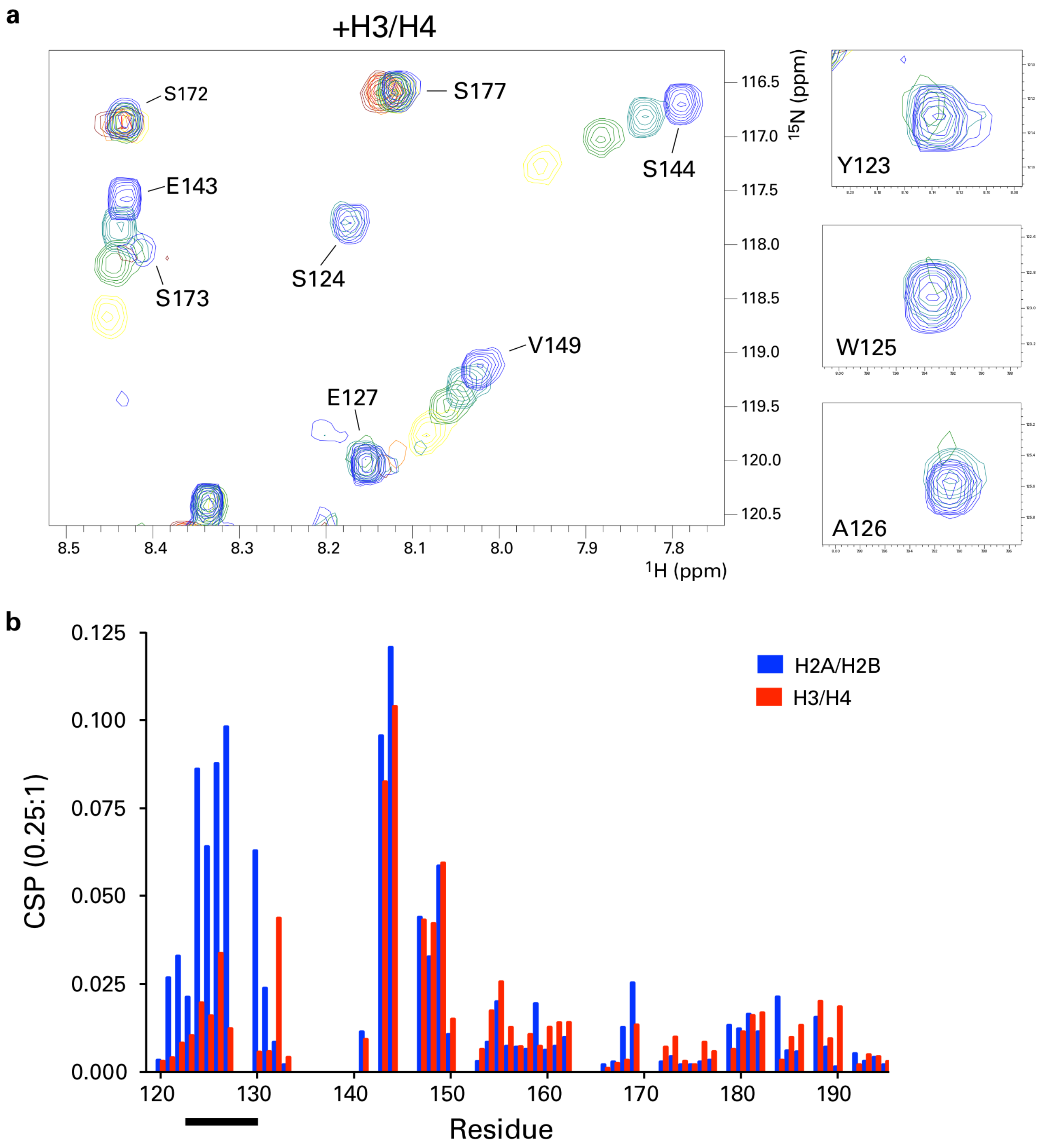


**Supplementary Figure 3: NMR Analysis of the Npm Tail Interactions with Histones H3/H4**

a. NMR-CSP example spectra of the Npm Tail binding to H3/H4.

b. CSP values at 0.25:1 molar ratio of H2A/H2B or H3/H4 dimers. Black bar indicates residues that disappear upon binding in both CSP experiments.

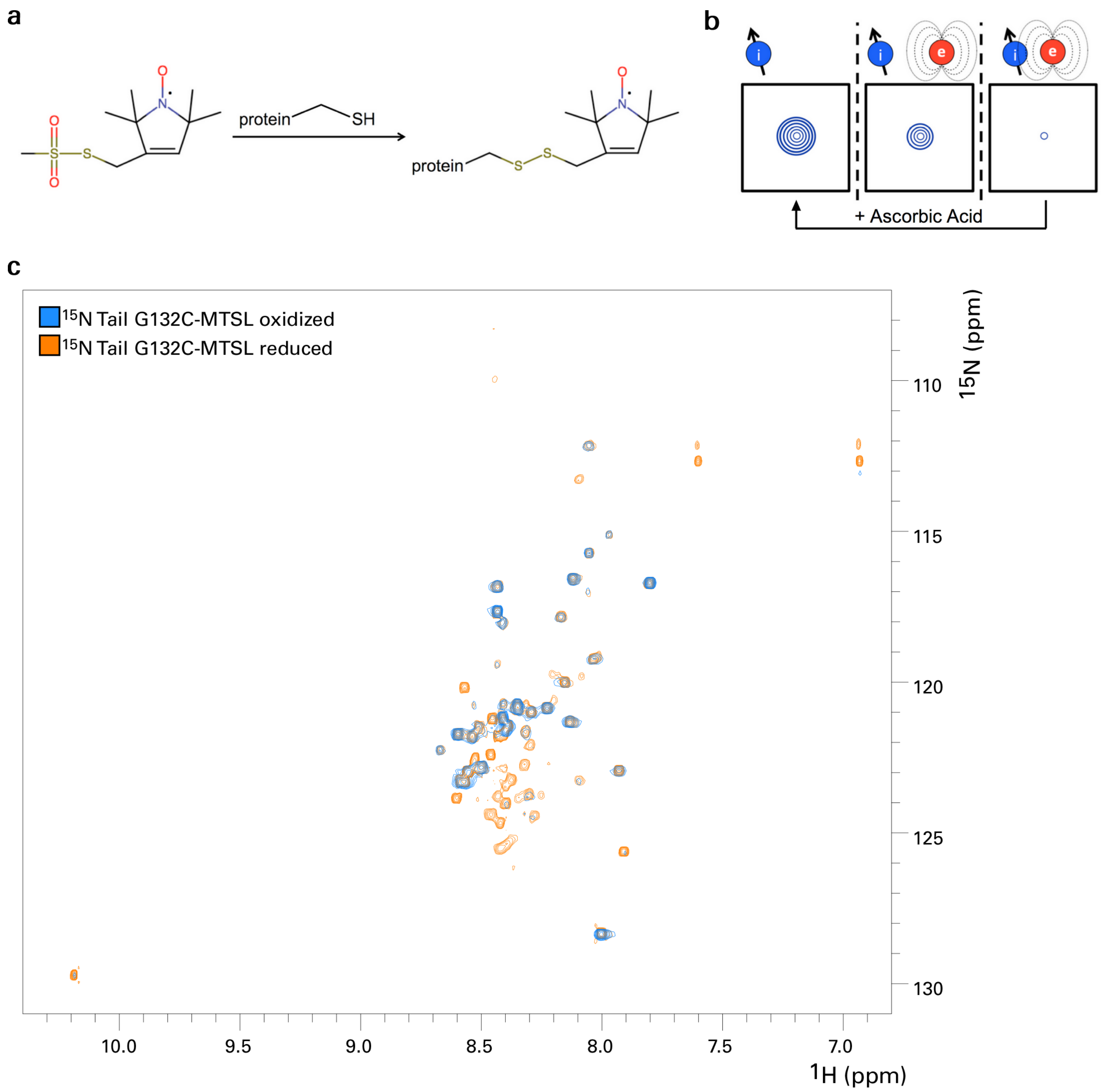


**Supplementary Figure 4: PRE-NMR analysis of Npm Tail Structure**

a. Chemical structure of the MTSL spin label and protein coupling mechanism.
b. Schematic of PRE-NMR experiments. Residues within ~25Å of the spin label show reduction in intensity due to free radical induced relaxation enhancement. Addition of ascorbic acid reduces the spin label leading to loss of PRE effects.
c. Representative HSQC spectra of the Tail labeled at G312C-MTSL in oxidizing conditions (blue) and after reduction with ascorbic acid (orange). Significant PRE effects were observed for many residues in the spectra.

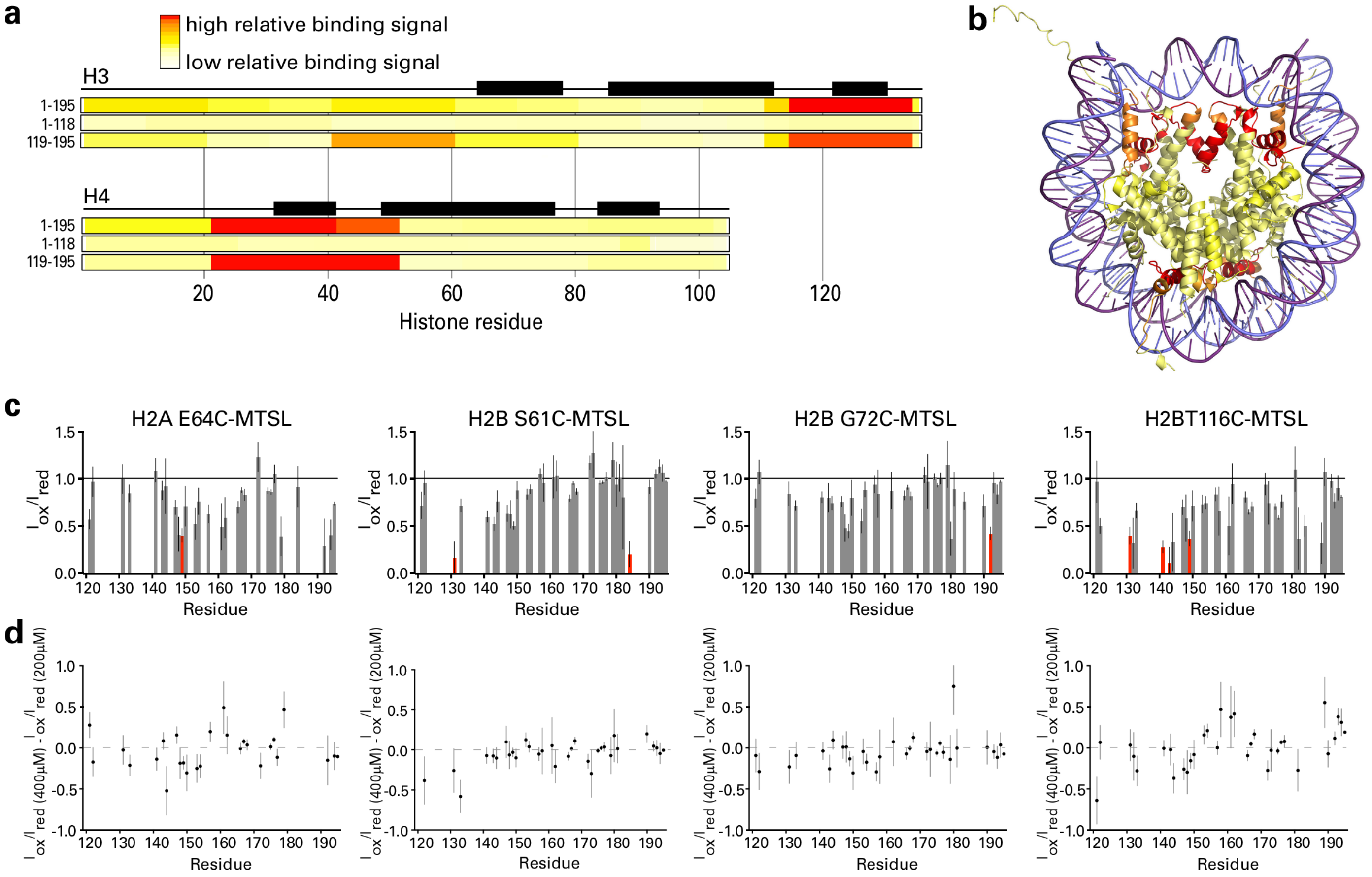


## Supplementary Figure 5: Histone Binding by the Npm Tail Domain

a. JPT histone peptide array result using 3 different Npm truncations probed against comprehensive H3/H4 peptides on a chip. Top=Full length Npm (1-195), Middle=Npm Core (1-118), Bottom=GST-tagged Npm Tail (119-195). Black boxes represent positions of $\alpha$-helices in the histone fold.

b. Results of histone peptide array (Figures 5A and S5A) mapped onto the structure of the nucleosome (PDB 1AOI). The Npm Tail binds mainly along the periphery of the nucleosome and at the H3/H4 dimer:dimer interface.

c. $I_{ox}/I_{red}$ graphs of the Npm Tail domain bound to H2A/H2B at 200μM concentration. 4 sets of intermolecular PRE effects derived from the complex. Error bars are inversely proportional to the propagated signal-to-noise ratio of individual resonances. Same coloring scheme as used in Figure 3.

d. Differences in $I_{ox}/I_{red}$ values from two datasets collected at different protein concentrations (400μM and 200μM) of the Tail bound to H2A/H2B. Error bars are propagated from the errors in the individual datasets.

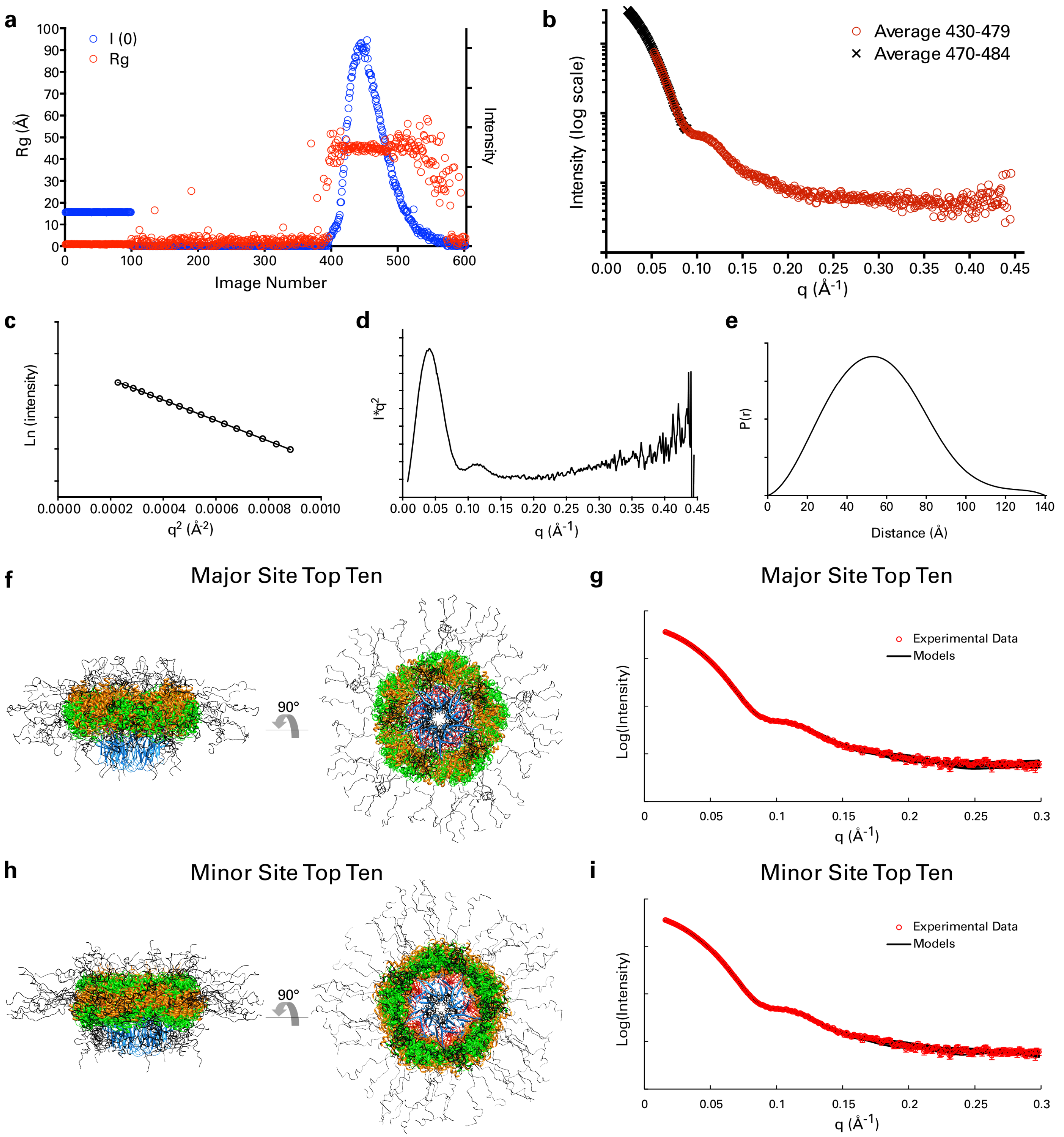


**Supplementary Figure 6: SAXS and Analysis of the Core+A2:H2A/H2B Complex**

a. $R_g$ and I(0) plots during SEC elution.
b. The scattering profile used for further SAXS analysis was generated by merging two average profiles corresponding to image number 430-479 and 470-484. The latter profile of q > 0.05 Å was scaled and merged with low angle part of the former profile (q < 0.09 Å).
c. Guinier plot of the Core+A2:H2A/H2B complex indicates no sample aggregation.
d. Kratky plot of the Core+A2:H2A/H2B complex indicates that the sample is well folded.

e. Pairwise distribution function, P(r), of the scattering profile indicates a largely globular sample.
f. Side and top view of top 10 models having low $\chi$ values (average $\chi$ = 1.643) using the best model from site #1 of A2:H2A/H2B complex as a starting structure (Figure 5D). Models were superimposed onto Npm Core domain whose position was fixed during modeling. Npm Core, A2, H2A and H2B are colored in blue, red, green and orange, respectively. Lacking fragments reconstructed by the program *CORAL* are colored in black.
g. Back calculated scattering curves of the top ten models using the best model from site #1 of A2:H2A/H2B complex (black lines) plotted with experimental data (red circles).
h. Side and top view of top 10 models having low $\chi$ values (average $\chi$ = 1.186) using the best model from site #2 of A2:H2A/H2B complex as a starting structure (Figure 5D). Models were superimposed onto Npm Core domain whose position was fixed during modeling. Npm Core, A2, H2A and H2B are colored in blue, red, green and orange, respectively. Lacking fragments reconstructed by the program *CORAL* are colored in black.
i. Back calculated scattering curves of the top ten models using the best model from site #2 of A2:H2A/H2B complex (black lines) plotted with experimental data (red circles).

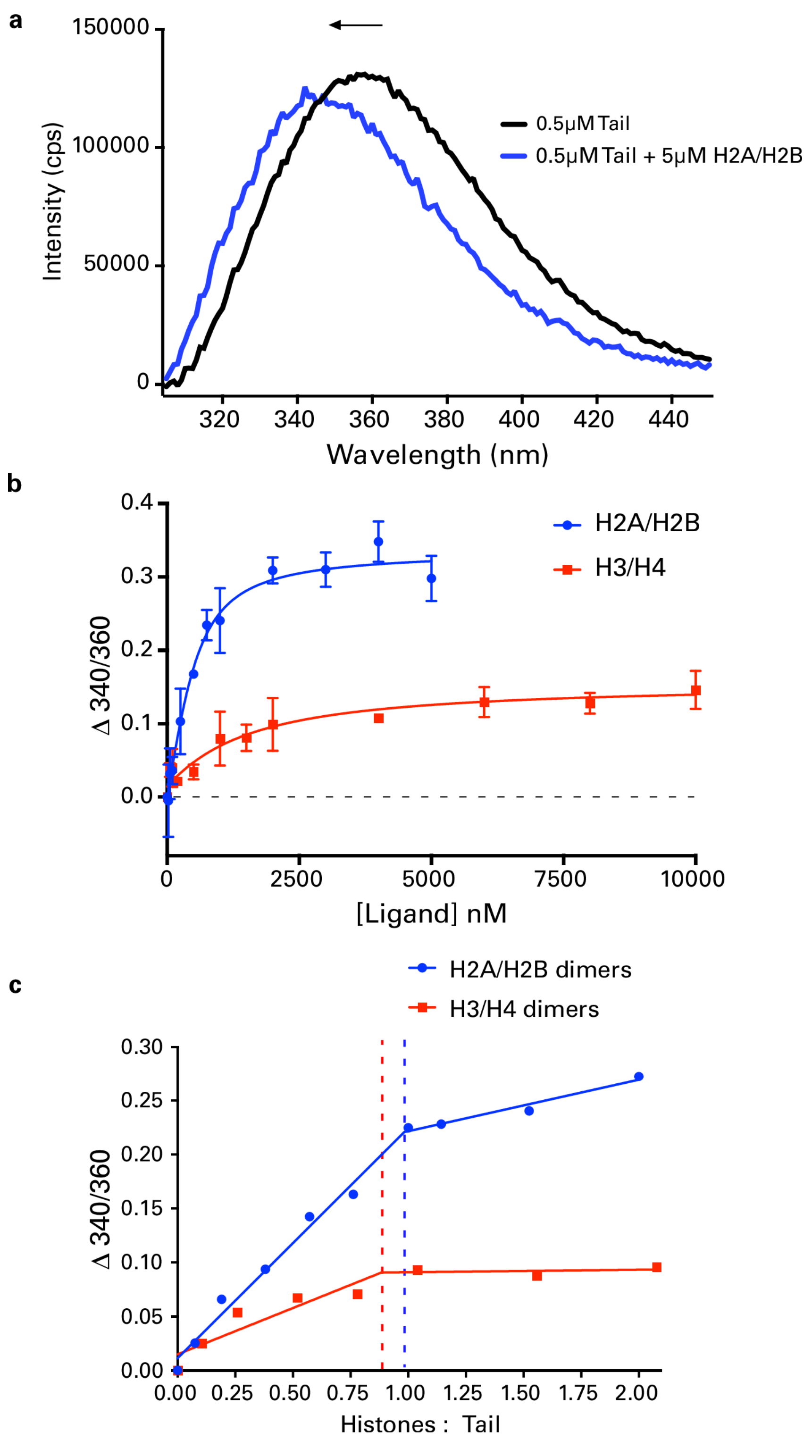


**Supplementary Figure 7: Tryptophan Fluorescence Analysis of Npm Tail Histone Binding**

a. Fluorescence emission spectra of the Npm Tail alone (black) and bound to H2A/H2B (blue). Peak shift was quantified by 340/360 intensity ratio (~0.8 in unbound to ~1.1 when bound).
b. Change in 340/360 ratio values (Δ340/360) upon binding to H2A/H2B or H3/H4. Data points fit to a single-site binding equation taking Npm Tail concentration into account. Binding of H3/H4 led to a less pronounced blue shift compared to H2A/H2B.
c. Stoichiometric titration of H2A/H2B or H3/H4 using 10μM of the Npm Tail domain. n = 0.98±0.07 for H2A/H2B dimers and n = 0.88±0.23 for H3/H4 dimers.

**Supplementary Table 1. SAXS data collection and scattering-derived parameters**

| | |
|---|---|
| *Data collection* | |
| Beamline | SSRL BL4-2 |
| Type of experiment | SEC-SAXS |
| SEC column | Superdex 200 PC3.2/300 |
| Sample volume | 90 μl |
| Sample concentration | 4.83 mg/ml |
| Beam defining slits size | 0.3 mm (H) x 0.3 mm (v) |
| Sample-Detector distance | 1.7 m |
| Wavelength | 1.127 Å (11 keV) |
| Beam current | 500mA (5 min top-off) |
| Exposure time | 1 sec |
| Temperature | 293 K |
| Sample cell size (quartz capillary) | 1.1 mm in diameter |
| | |
| Guinier analysis | |
| $q*R_g$ limit* | 0.7-1.3 |
| I(0) | 2875.5 +/- 14.8 |
| $R_g$ | 43.9 +/- 0.284 Å |
| | |
| P(r) and Porod volume estimation | |
| Software | Primus/GNOM |
| q range* | 0.02-0.18 $Å^{-1}$ |
| I(0), real space | 2822 +/- 8.133 |
| $R_g$, real space | 43.16 +/- 0.110 Å |
| $D_{max}$ | 140 Å |
| Porod volume | 402000 $Å^3$ |
| Calculated pentameric Mw (kDa) from sequence | 219.7 kDa |
| | |
| *Rigid body modeling* | |
| Software | CORAL |
| q range* | 0.016-0.3 $Å^{-1}$ |
| Number of runs | 50 |
| | |
| *ab initio* modeling | |
| Software (ab initio modeling) | DAMMIF |
| Software (averaging) | DAMAVER |
| | |
| q range* | 0.02-0.18 $Å^{-1}$ |
| Number of runs | 30 |
| Normalized Spatial Discrepancy (NSD) | 0.846 +/- 0.248 |

*$q = 4\pi\sin(\theta)/\lambda$, where $2\theta$ is the scattering angle.
